# Genomic Evolution of Porcine Reproductive and Respiratory Syndrome Virus (PRRSV) Isolates Revealed by Deep Sequencing

**DOI:** 10.1371/journal.pone.0088807

**Published:** 2014-04-03

**Authors:** Manreetpal Singh Brar, Mang Shi, Raymond Kin-Hi Hui, Frederick Chi-Ching Leung

**Affiliations:** 1 School of Biological Sciences, The University of Hong Kong, Hong Kong, China; 2 Sydney Emerging Infections & Biosecurity Institute, School of Biological Sciences and Sydney Medical School, The University of Sydney, Darlington, Australia; Centro Nacional de Microbiología - Instituto de Salud Carlos III, Spain

## Abstract

Most studies on PRRSV evolution have been limited to a particular region of the viral genome. A thorough genome-wide understanding of the impact of different mechanisms on shaping PRRSV genetic diversity is still lacking. To this end, deep sequencing was used to obtain genomic sequences of a diverse set of 16 isolates from a region of Hong Kong with a complex PRRSV epidemiological record. Genome assemblies and phylogenetic typing indicated the co-circulation of strains of both genotypes (type 1and type 2) with varying Nsp2 deletion patterns and distinct evolutionary lineages (“High Fever”-like and local endemic type). Recombination analyses revealed genomic breakpoints in structural and non-structural regions of genomes of both genotypes with evidence of many recombination events originating from common ancestors. Additionally, the high fold of coverage per nucleotide allowed the characterization of minor variants arising from the quasispecies of each strain. Overall, 0.56–2.83% of sites were found to be polymorphic with respect to cognate consensus genomes. The distribution of minor variants across each genome was not uniform indicating the influence of selective forces. Proportion of variants capable of causing an amino acid change in their respective codons ranged between 25–67% with many predicted to be non-deleterious. Low frequency deletion variants were also detected providing one possible mechanism for their sudden emergence as cited in previous reports.

## Introduction

Porcine reproductive and respiratory syndrome (PRRS) is a prominent swine disease that first emerged towards the end of the 1980s characterized by complications in gestation or reproductive failure in pregnant sows, neonatal loss, and affliction of the respiratory system in young piglets [1]. Reports in North America [2] were shortly followed by incidences across Europe [3]–[6]. Gradually hog rearing Asian nations were impacted as well [7]–[10]. The disease continues to cause immense economic loss [11]. The causative agent, PRRS virus (PRRSV), is a single-stranded positive sense RNA virus with an approximate genome size of 15 kb.

Comparisons of prototype isolates from Europe (Lelystad virus; LV) and North America (ATCC VR2332) revealed stark genetic difference [12], [13] which was supported at the antigenic level as well [14]–[16]. This established the designation of isolates genetically closer to LV as of European genotype (EU type or type 1) and those similar to VR2332 as of North American genotype (NA type or type 2). More recently, high resolution phylogenetic studies using large scale sequence data representing current known genetic diversity also support clear division into two genotypes with no new or intermediate genotype detected though marked diversity exists within each genotype [17], [18]. From this genetic diversity, PRRSV has been documented to periodically re-emerge in particularly heightened virulent forms causing tremendous loss to the swine industry in a short time span [19]–[21].

Evolutionary mechanisms at the micro level responsible for the described salient features of PRRSV genetics primarily include a relatively high mutation rate and recombination. The former is a common trait in RNA viruses due to the lack of proofreading capability of the inherent viral RNA polymerase. The error-prone replication together with quick replication kinetics gives rise to a mutational cloud of variants known as quasispecies [22]. However, no thorough investigative work at the whole genome level on characterizing this mist of closely resembling variants following PRRSV infection has been carried out. Recombination within each genotype of PRRSV has been demonstrated as a possibility for viral diversification in cell culture based studies [23]–[25]. To date, knowledge of natural circulating recombinants in the field is scarce and even less is known about their genetic architecture that provides biological fitness to compete with parental strains.

To these ends, we applied massively parallel deep sequencing technology to PRRSVs from both cell culture propagated and naturally infected swine tissue. All isolates were from the Hong Kong SAR, a region for this study for two main reasons. First, farms are mostly cluttered close to each other in one restricted geographic region with a history of multiple circulating strains within single farms. This provides opportune incidence for natural recombination resulting from host co-infection. Second, Hong Kong (HK), though having its own system of governance, sits at the southern tip of China which has experienced highly pathogenic bouts of PRRSV recently [20], [26] but not much is known of locally circulating viral diversity or its evolutionary relatedness to the mainland. Our work provides important perspectives in terms of: (1) Genomic characterization of local PRRSV diversity including naturally circulating recombinants; (2) the first ever quantitative profiling of polymorphic sites across consensus genomes derived from quasispecies based nucleic acid deep sequencing; (3) impact assessment of detected SNPs on protein diversity and functionality.

## Materials and Methods

### Viruses

All samples were obtained from the Agricultural, Fisheries and Conservation Department of Hong Kong (HK) which represented local field isolates. For samples with PRRSV in low titer, samples were homogenized in PBS solution, centrifuged, and passed through a 0.22 µM filter. The filtrate was then used as inoculum for MARC-145 cell culture. Upon CPE, only culture supernatant was collected and stored until further use. The passage number for these viruses on MARC-145 cells did not exceed two. For lung tissue viruses, the above process was repeated except the filtrate after homogenization was used directly for nucleic acid extraction.

### PRRSV amplicons and pyrosequencing

Total nucleic acid was extracted from cell culture supernatant or tissue filtrate samples using the QIAamp Viral RNA Mini Kit (Qiagen). 10 µl of each eluate was used for cDNA synthesis with SuperScript II Reverse Transcriptase (Life Technologies) using random hexamers (Life Technologies) under conditions outlined by the manufacturer. Four genotype specific PCR reactions (each of total volume 25 µl) per strain spanning the PRRSV genome (primers given in Table S1 in File S1) were set-up using the High Fidelity^PLUS^ PCR System (Roche) inputting 1.5 µl of synthesized cDNA as template. Volume of PCR constituents and cycling settings (thermal profile A, 35 cycles) were in accordance with the product documentation. Size of target PCR products was confirmed by electrophoresis on a 1% agarose gel. PCR products were excised and gel purified using the PureLink Quick Gel Extraction Kit (Life Technologies) and quantified using the Quant-iT PicoGreen dsDNA Assay Kit (Life Technologies) with readings taken on the FLUOstar OPTIMA F fluorometer (BMG Labtech GmbH). The four amplicons for each sample were pooled in an equimolar manner and used for library construction using the Rapid Library Preparation Kit (454 Life Sciences) along with a unique MID adaptor. A group of eight such libraries, each quantified with the Quant-iT PicoGreen dsDNA Assay Kit (Life Technologies), were pooled in an equimolar manner for a single emulsion-PCR and sequencing reaction using titanium chemistry on the 454 GS Jr platform (454 Life Sciences). Sequencing procedures followed instructions by the manufacturer. Raw sequence data has been deposited under the NCBI SRA accession SRP033216.

### Genome assembly and variant calling

Post-sequencing, sample-specific reads were demultiplexed based on MIDs. Reads were quality trimmed using a cutoff of a mean quality score of 20 in a 50 nucleotide window. Primer sequences were removed as well before utilizing GS Reference Mapper (454 Life Sciences) to construct consensus genomic sequences using genotype specific prototype strains as references wherein the dominant nucleotide was taken in the case of polymorphic sites. Genome sequences have been deposited into GenBank under the accession numbers KF287128-KF287143. Several criteria were adopted in accepting minor single nucleotide polymorphisms (SNPs): (1) at least two non-duplicate reads that differ from the consensus sequence; (2) a minimum of five bases flanking both sides of the differing site; (3) at least one sense and one antisense read; (4) no more than a five-fold difference between the number of sense and antisense reads; additionally, for indels: (3) only those of a length that was a multiple of three. This last criterion excluded indels either resulting from sequencing errors or viral replication inaccuracies causing frameshift mutations that would in any case prevent such occurrences from becoming fixed in the viral population.

### RT-PCR and Sanger sequencing

To confirm the observed Nsp2 deletions were not the result of assembly errors. Genomic RNA was reverse transcribed by SuperScript II Reverse Transcriptase (Life Technologies) with the cDNA used in PCR employing Plantinum Taq Polymerase (Life technologies) involving primers (available upon request) flanking deletion sites as predicted by 454 read assemblies. PCR products were sequenced directly using BigDye chemistry on the ABI 3130xl platform. Partial 5′ and 3′ UTRs as well as gaps in the genome of viral strain #8 (due to comparatively lower sequencing depth) were similarly determined.

### Phylogenomic analysis

Genomic sequences from the present study were combined with others downloaded from GenBank. Genotype specific alignments were performed using MUSCLE v3.7 [27] under default settings with minor manual adjustment afterwards. Maximum likelihood inference for tree construction implemented in MEGA5 [28] was used under a general time-reversible (GTR) model with discrete gamma distributed rate variation among sites (Ī4) and a proportion of invariable sites (I). Exact parameters employed and assignment of lineage level genotyping has been described elsewhere [18].

### Bioinformatic and statistical analyses

All statistical tests were performed in R [29]. Strain-specific and inter-strain genomic traits were visually summarized using circos [30]. Screening for potential recombinant strains was performed using RDP [31], GENECONV [32], BOOTSCAN [33], MAXCHI [34], CHIMAERA [35], SISCAN [36], LARD [37], and 3SEQ [38] collectively implemented in RDP3 [39]. Recombination events were only considered significant when confirmed by at least four of the above methods together with phylogenetic support. Predictive effects of nonsynonymous SNPs on protein structure were ascertained using PROVEAN [40] and SNAP [41] tools under default settings.

Genetic complexity arising from viral quasispecies at each genomic site was assessed by the normalized Shannon entropy index which was calculated using the formula:
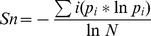
where *N* is the total number of reads analyzed at a site and *p_i_* is the frequency of each nucleotide variant at that site. *Sn* ranges from 0 (no complexity) to 1 (maximum complexity). The mean viral complexity in each genome or ORF was determined by averaging the normalized Shannon entropy at all sites in either the respective genome or ORF.

## Results

### Multiplexed whole genome sequencing of HK PRRSV strains

**Figure 1:**
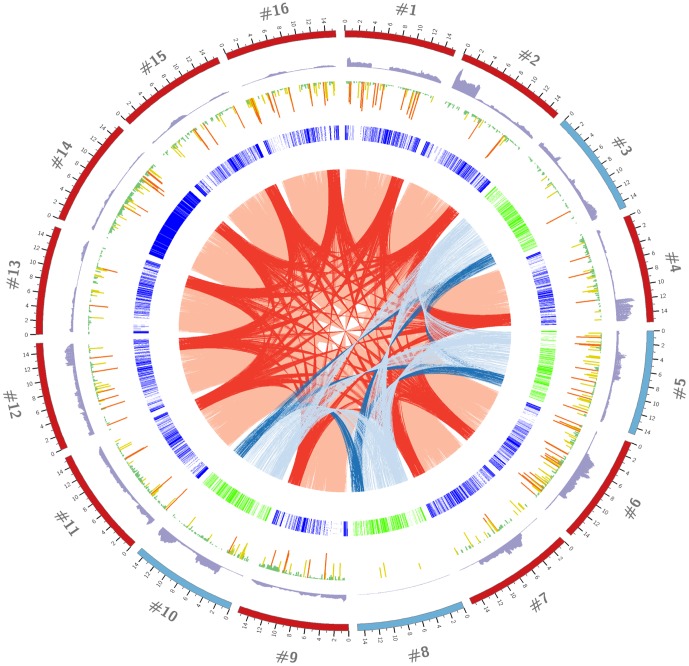
Deep sequencing of HK PRRSV strains. Shown from the periphery to the centre of the figure for each strain is strain ID, genome ideogram (blue for type 1; red for type 2), sequencing depth across the genome, minor variants detected across the genome with at least 1% frequency (<10% in green, > = 10% in yellow, > = 30% in red), amino acid comparisons (green for type 1; blue for type 2) with reference strains (LV for type 1; ATCC VR2332 for type 2) for individual ORFs across the genome (non-conformity represented by white streaks), and pairwise aligned amino acid differences between sequenced strains for individual ORFs (non-structural ones in light shade; structural ones in dark shade) across the genome (blue for type 1; red for type 2).

The bulk of past studies on characterizing PRRSV diversity or evolution have been restricted to mostly one or two viral genes only, mainly ORF5 and ORF7. To expand knowledge of genomic diversity, we applied deep sequencing to a total of 16 type 1 and 2 PRRSV samples obtained from either cell culture supernatant or swine lung tissue. On average 99.66%±0.35% (s.d.; n = 16) quality filtered sample specific reads were incorporated into final assembly results in which 14 out of 16 instances produced single contigs. These contig(s) spanned on average 96.50%±1.35% (s.d.; n = 16) of reference genomes (LV for type 1; ATCC VR2332 for type 2). A summary of individual sample information, assembly metrics, and genomic comparisons with prototype isolates is provided in Table 1. Pairwise comparisons at the nucleotide level for all isolates with respect to their genotype specific reference strains revealed significant genomic divergence from these initial PRRSV isolates first characterized in the early 1990s. This nucleotide diversification, measured by p-distance, was more pronounced in type 2 strains (mean = 0.103; n = 12) than type 1 strains (mean = 0.088; n = 4). Similarly, inter-strain comparisons (type 1 mean = 0.054; type 2 mean = 0.107) indicated high genetic diversity in local circulating strains with varying patterns of Nsp2 deletions. The impact of this genetic diversity on protein sequence variability was high with respect to both reference strains and local isolates (Figure 1). An exception was strain #14 which likely represented a derivative of the vaccine strain Ingelvac MLV (blastn search: 99% identity) which in turn is a modification of the type 2 reference strain [42].

**Table 1:** Summary of sample information, assembly-related statistics, and genomic features of sequenced PRRSV strains.

Sample ID	Isolation date	Sample nature#	Genotype	No of reads+	No of contigs	Reads captured in contig(s)	Coverage∧ (%)	Average sequence depth//	Genomic divergence from reference strains (p-distance)	Nsp2 deletions*
#1	2003	CCS	Type 2	15939	1	15898	96.46	312	0.098	Δ314–439,482–486, 832–848
#2	2003	CCS	Type 2	19083	1	18993	96.46	389	0.119	Δ469–518, 813–848
#3	2003	CCS	Type 1	15320	1	15275	98.16	319	0.086	Δ180,417–420
#4	2003	CCS	Type 2	17121	1	17043	96.46	349	0.101	None
#5	2004	CCS	Type 1	14700	1	14671	98.16	306	0.089	Δ180, 417–420
#6	2004	CCS	Type 2	22731	1	22696	96.46	462	0.126	Δ481, 813–848
#7	2004	CCS	Type 2	15650	1	15625	96.46	333	0.100	Δ314–439, 482–486, 832–848
#8	2004	CCS	Type 1	290	7	290	92.11	7	0.087	Δ180, 288–294, 348–376, 417–420
#9	2004	CCS	Type 2	11864	1	11796	96.46	223	0.127	Δ813–848
#10	2004	CCS	Type 1	17943	2	17902	98.11	368	0.088	Δ180, 417–420
#11	2004	CCS	Type 2	15929	1	15892	96.46	328	0.123	Δ813–848
#12	2004	CCS	Type 2	20636	1	20583	96.46	418	0.103	None
#13	2005	CCS	Type 2	7335	1	7311	96.46	141	0.096	Δ813–848
#14	2004	CCS	Type 2	5595	1	5584	96.46	108	0.003	None
#15	2004	SLT	Type 2	5400	1	5391	96.46	110	0.118	Δ813–848
#16	2004	SLT	Type 2	4205	1	4140	96.46	81	0.127	Δ813–848

# : cell culture supernatant (CCS); swine lung tissue (SLT).

∧ : coverage estimated using prototype isolates for type 1 (LV) and type 2 (ATCC VR2332) PRRSV as references.

*: positions of amino acid deletions given are based on the Nsp2 protein from prototype isolates of type 1 (LV) and type 2 (ATCC VR2332) PRRSV.

+ : number obtained after quality filtering procedures detailed in the methods section.

// : depth calculation takes into account duplicate reads as well.

### Co-circulation of strains with varied evolutionary lineages and recombination histories

**Figure 2:**
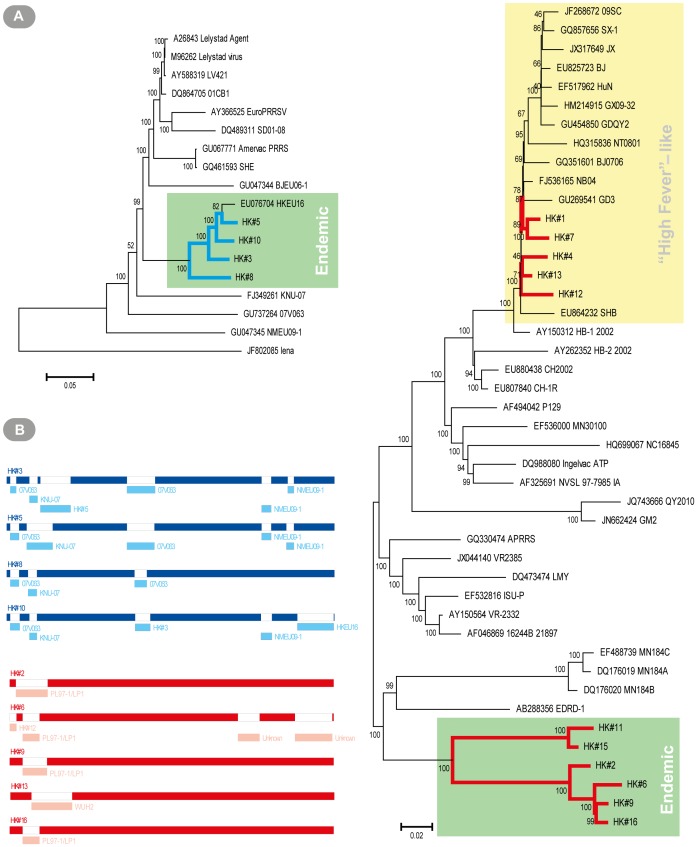
Phylogenomic and recombination characterization of HK PRRSV strains. (A) Genotype-specific (type 1 left; type 2 right) phylogenies of HK and globally sequenced PRRSV genomes. Type 1 strains form a monophyletic clade separate from isolates of any other country or region. Type 2 strains classified with either previously known local as known as “endemic” diversity or the “High-Fever”-like variants isolated during virulent outbreaks of PRRS in China. (B) Mapped recombination breakpoints and potential minor parental-like strains of mosaic HK PRRSV strains. Unknown designation (for a missing parental-like strain) was assigned when a recombination event was characterized from an alignment involving only one parental-like strain and the recombinant.

Phylogenetic and recombination analyses in context of our previously established genotyping system [18] was performed to elucidate the significant genetic diversity seen amongst local isolates. From an evolutionary standpoint, all of the HK type 1 strains were closely-related forming a monophyletic group whereas type 2 strains were classified to either “endemic” diversity or the “High-Fever”-like group of isolates that widely circulated on the mainland (China) during virulent outbreaks of PRRS (Figure 2A). Strains belonging to the latter group represented introduced PRRSV incidents to Hong Kong explaining the genetic disparity with “local or endemic” isolates. Moreover, majority of the sequenced isolates registered recombination breakpoints as well with type 1 strains being much more mosaic in nature compared to the type 2 strains (Figure 2B). A complete description of the location of breakpoints, parental-like strains, and support from the different detection methods is given in Table S2 in File S1 and Figure S1 File S1 for all recombination events. The topology of the phylogeny, potential minor parental-like strains, and breakpoint locations in the genome suggested most of the recombination events were not independent but took place in a common ancestor followed by successful circulation of descendant recombinants in the field. There was also evidence of recombination in local isolates, in the cases of strains #3, #6, and #10, with other strains of their same genotype but no indication of genomic exchange between endemic and High Fever-like strains.

### Nucleotide variants across consensus PRRSV genomes from intra-sample viral populations

Quasispecies variation has been speculated to play an important role in PRRSV infection and persistence. We proceeded to characterize these viral variants at the genome level post-infection in cell culture and swine lung (PRRSV has a predilection for alveolar macrophages [43]) since no such work exists. A detailed summary of characterized minor variants for each viral strain is presented in Table 2. Due to the low sequencing depth obtained for strain #8, it was left out of further variants-related analyses. For the approximately 15 kb captured region of the PRRSV genome, the percentage of variant sites passing screening criteria was in the single digits (0.56–2.83%). The frequency of these variants across each genome is shown in Figure 1. The median minor variant frequency ranged between 0.88–17.42% across different genomes. However, it should be noted that the estimates of polymorphic sites and minor variant frequencies are not definitive as these will most likely vary with experimental or sampling conditions as well as sequencing depth. Nonetheless, the variants that were detected with the given frequencies underscores the need for a high depth of sequencing to capture many low abundance variants which might otherwise be missed in traditional Sanger sequencing or low depth next generation sequencing.

**Table 2:** Summary on the nature, incidence, and impact on codons of minor variants across consensus genomes of HK PRRSV strains.

Strain ID	Non-coding region variants	Coding region variants										
		Transitions		Transversions				InDels	Overall codon diversity (%)*	Nonsynonymous variants (%)+	Nonsynonymous codon variants (%)∧	Mean *Sn* (×10^−4^)
		A↔G	C↔T	A↔T	C↔G	A↔C	G↔T					
#1	0	108	143	4	1	2	4	0	5.04	57.25	56.46	4.03
#2	0	121	170	13	5	8	20	0	6.35	45.99	45.13	3.62
#3	0	68	98	1	3	3	4	0	3.40	63.84	63.33	1.74
#4	0	101	152	5	1	2	3	0	4.98	66.67	64.03	3.62
#5	1	64	83	4	1	2	6	0	3.16	46.25	45.93	4.11
#6	0	165	235	11	2	7	4	0	7.81	58.02	60.10	5.70
#7	0	87	93	4	0	2	6	0	3.70	53.13	53.23	2.22
#8	0	0	1	0	0	0	0	0	0.02	0	0	/
#9	1	140	180	16	6	9	12	1	6.47	52.75	51.67	8.81
#10	1	94	165	7	2	5	3	0	5.23	48.19	48.91	5.36
#11	0	103	143	9	1	5	10	0	5.39	53.51	53.96	4.65
#12	1	134	162	4	2	8	7	0	6.14	48.26	47.35	3.34
#13	0	53	77	3	2	3	1	1	2.70	51.43	51.06	3.04
#14	0	129	187	15	3	12	18	1	6.89	25.14	26.55	12.72
#15	0	30	46	1	1	0	3	0	1.59	48.15	48.15	2.25
#16	1	31	65	5	0	4	5	0	2.01	43.64	46.15	8.16

*: percentage of codons (out of the total number of codons) in the coding region with at least one minor variant.

+ : percentage of minor variants (out of the total number of variants) capable of causing an amino acid change in their respective consensus codons.

∧ : percentage of codons with variants (out of the total number of codons with variants) capable of causing an amino acid change in their respective consensus codons.

*Sn*: Shannon entropy.

**Figure 3:**
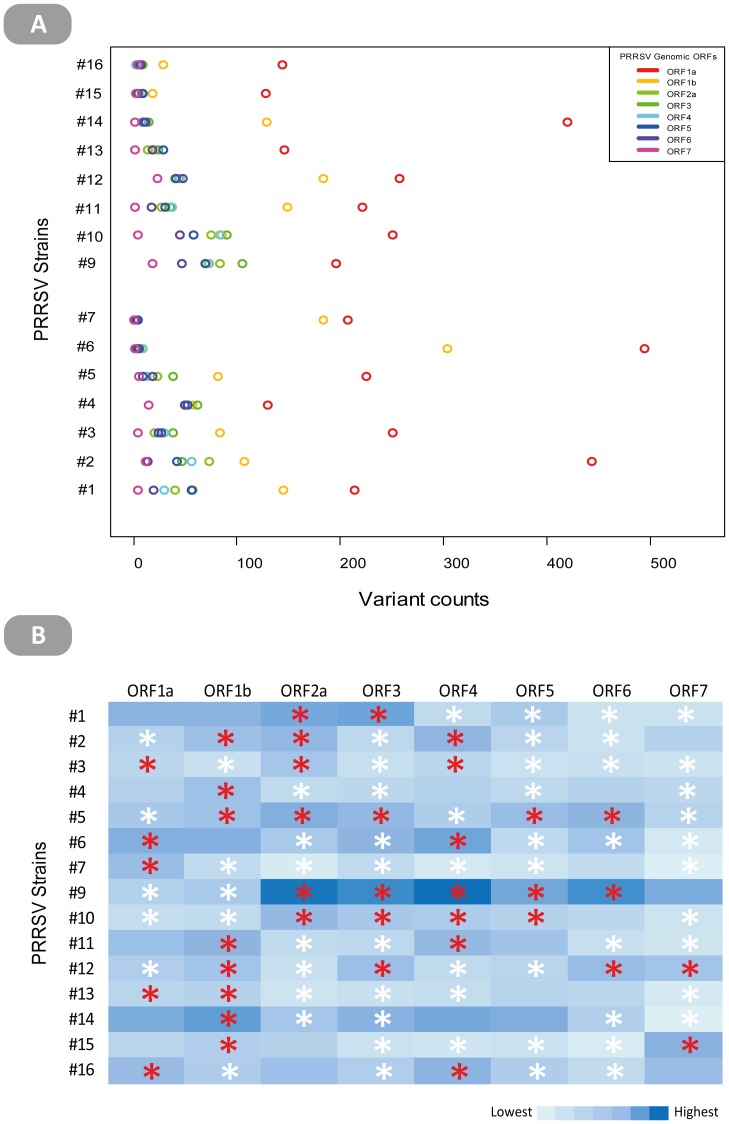
Genetic complexity arising from nucleotide variants across ORFs of different PRRSV strains. The quantitative measure of mean normalized Shannon (*Sn*) entropy accommodated for differences in sequencing depth and variable ORF sizes. Open (**○**) and closed (•) circles represented ORF and genomic mean *Sn* entropies respectively.

Since the sequenced genomic regions partially or wholly missed the 5′ and 3′ untranslated regions and due to the overlapping nature of coding ORFs, this meant very few variants were detected in non-coding regions. The bulk of minor variants were located in both single and overlapping ORF areas. Figure 3 gives a quantitative measure of genetic complexity in different ORFs arising from coding region variants for the 15 strains. An important question for consideration was whether minor variants structured or concentrated in particular patterns or regions of the PRRSV genome. Since PRRSV ORFs are unequal in size and sequence depth varied across genomes, the mean normalized Shannon entropy (*Sn*) for both individual ORFs and the entire genome was calculated taking into account these inequalities. Skewness in the mean *Sn* of ORFs compared to their genomic mean differed to a great extent between strains (minimal in strains #3 and #4 and maximal in strains #9 and #14). Ranking the *Sn* means of ORFs across isolates did not reveal any decisive pattern(s) of structured genetic complexity. However, in most cases, a structural ORF tended to have the highest mean *Sn*. Nonetheless, no generalizations can be made from this initial study with limited sample size.

There is some controversy surrounding the use of modified live vaccines (MLVs) in the field with regard to the possibility of reversion to virulence [44], [45]. This controversy aside, the biological forces acting on or influencing vaccine evolution post-administration also need to be studied better. Strain #14 which was found to be a MLV-like virus exhibited a large repertoire of variant sites of which most were localized in ORF1a only with very few variable sites recorded in the structural ORFs. To assess whether reversion to virulence was possible, we surveyed minor variants at sites differing between ATCC VR2332 and strain #14 to see if captured nucleotide diversity allowed this. In total, 46 sites differed between the two strains within the pyrosequenced region. Of these, 18 sites either had a minor variant in agreement with the wild-type strain or the differing nucleotide imposed no impact on the cognate translated amino acid.

### Nature of minor variants and their potential for proteomic consequences

Minor variants primarily consisted of nucleotide transitions with C↔T counts always higher than A↔G counts (Table 2). Collectively, nucleotide transitions outnumbered transversions by approximately 2–10 fold depending on the viral strain. No particular one type of transversion was predominant across isolates. The distribution of minor variants across individual codons of different ORFs to produce codon variability at those sites was termed codon diversity. Cell culture strains exhibited higher codon diversity (2.70–7.81%) compared to lung tissue counterparts (1.59–2.01%). In terms of ORFs, codon diversity, at its highest, was more than double in structural constituents (29.61%) than that in nonstructural ones (11.6%).

Genomic diversity among quasispecies virions has the potential for proteomic ramifications in such populations. Accordingly, an assessment of the impact of characterized nucleotide minor variants on protein diversity was made as well. The proportion of single nucleotide variants capable of causing a change in the amino acid residue of respective codons for each strain ranged between approximately 25–67% (Table 2). In terms of codons with minor variants, the percentage exhibiting translational changes was in a similar range.

**Figure 4:**
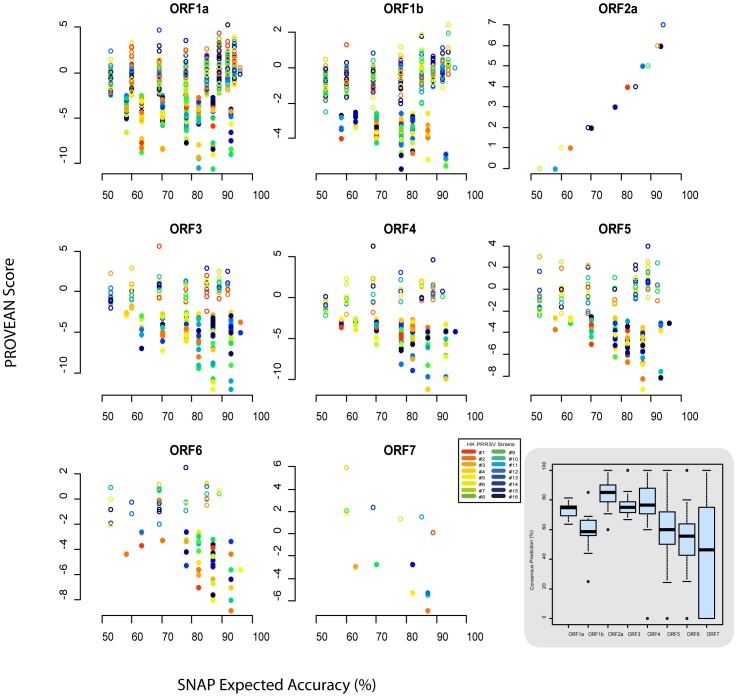
Predicted effect of minor nonsynonymous variants on the protein structure of different PRRSV ORFs. Predicted effects were classified as being either neutral (**○**) or non-neutral (•) only when such effects were independently confirmed by PROVEAN and SNAP tools. Variants which had differing predicted effects by the two software tools were excluded. For PROVEAN, “neutral” designation was assigned when a score higher than −2.5 was obtained and “non-neutral” when lower. Confidence of predictions made by SNAP is indicated by the expected accuracy score generated by the tool. Results are summarized based on individual ORFs (first eight panels) and viral strains (color legend, panel 8). The distribution of consensus predictions (out of total predictions) by the two programs across ORFs of different strains is summarized in the last panel as boxplots (bottom right).

Aside from the consequence on the protein sequence, in silico assessments of the impact of nonsynonymous variants on protein structure were made using two independent predictive tools [40], [41]. Results only from unanimous predictions is summarized based on the confidence of predictions in Figure 4. The median value for consensus predictions (out of total predictions) amongst the different ORFs was at least ∼50–100%. The ORF-to-ORF variability in predictions can be ascribed to the lack of control over compilation of comparative sequence database per query in each tool and the heterogeneity in the number of comparative sequences available for each protein in public databases. Predictions were classified as being either neutral (tolerable impact) or non-neutral (deleterious or gain-of-function impact). Only with respect to ORF1a (6–46%) and ORF1b (0–10%) was there a clear discernible pattern of non-neutral point mutations being in a minority for each strain. For all other ORFs, dominance of either neutral or non-neutral mutations was variable over a wide range. Collectively, the present results are the first to provide insight into the nature and effect of minor variants arising from PRRSV quasispecies. However, the various quantitative estimates should not be taken as rigid estimates as they are likely to vary due to technical (e.g. experimental conditions and sequencing depth) and biological (e.g. viral replication kinetics and selective pressures) reasons.

One conspicuous genetic feature of PRRSV is the circulation of deletion variants in the field. Although these deletions have been reported in different ORFs, most pertain to the Nsp2 region but can be variable in pattern [46]–[48] and in some cases coinciding with particularly virulent outbreaks of PRRS [20], [49]. However, in at least one such case, the Nsp2 deletion does not appear to be the primary contributor to heightened pathogenicity [50]. As such, not much is known of the role or origin of such circulating mutants. To our knowledge, we report here for the first time the detection of co-existing low frequency deletion mutants in “singular” propagated non-deletion strains. A few strains (, #9, #13, and #14) harboured these low level deletion variants in different regions of the viral genome (Table 3). Only one deletion pattern per strain was observed with only single amino acid deletions in two out of three instances. An exception was in strain #14 which showed a larger nine-amino-acid deletion located in the Nsp2 region.

**Table 3:** Summary features of deletions detected in minor variants of a subset of HK PRRSV strains.

Strain	Genomic position	Region	Affected sequence		Variant Frequency (%)
			Nucleotide	Amino Acid	
#9	12807–12809	ORF2a_ORF3*	ATT/−	I/−	6.17
#13	3799–3801	ORF1a	GGT	G/−	28.57
#14	2713–2715	ORF1a	CCGGTTTCATTAGGCGGCGATGTCCCT/−	PVSLGGDVP/−	7.89

*: Deletion falls in the overlapping region of adjacent ORFs affecting both.

## Discussion

Although the number of swine farms in Hong Kong is small, their localization in one particular area and close geographic proximity to China meant local herds were exposed to multiple PRRSV genotypes and strains with varied evolutionary history. Both less virulent and “High fever”-like strains of type 2 genotype were identified. Recombination also appeared to have played an important role in shaping local circulating diversity though field reports on PRRSV recombinants in general is relatively rare. Almost all (8 out of 9) of the recombinant strains were typed to lineages (“endemic”, Figure 2A) to which HK PRRSV diversity has historically belonged to. Whether this is attributable to conditions specific to Hong Kong that provide opportune incidence for recombination to occur or the isolates represented successful recombinants from a few rare events cannot be determined presently. Nonetheless, the generation of mosaic strains poses additional concern in deciding a vaccination regimen aside from the significant inter-strain genetic diversity that was observed.

Using high sequencing depth revealed, for the first time, a quantitative account of polymorphic sites across consensus genomes resulting from quasispecies. Pronounced heterogeneity was seen in genetic complexity, due to minor variants, among the same ORFs of different strains. Even within individual samples, the skewed distribution of variants across the genome suggested factors other than polymerase infidelity alone influenced the location and maintenance of variable sites in quasispecies. While natural selection is bound to one of these factors other contributors could be the fitness of a particular strain to its environment (swine vs. cell culture), evolutionary history (recombinant vs. non-recombinant), or genetic modification (MLV-like isolates).

The MLV-like strain #14, though “attenuated”, maintained a swarm of minor variants. This was not surprising since MLV itself is still a live vaccine. Genomic comparisons between the wild-type isolate ATCC VR332 and RespPRRS MLV indicate approximately a third of all nucleotide differences to be located in ORF1a, a hotspot region for strain #14 minor variants as well, which prompted us to examine the question of reversion to virulence vis-à-vis SNP diversity. Based on the captured diversity, a complete reversion to parental form did not appear possible. However, a large share (∼39%) of nucleotide differences between strain #14 and ATCC VR2332 were either directly resolvable (wild-type and minor variant conformity) or had no proteomic consequences. Since all samples in the present study were collected at a single time point only, it is open to speculation whether genetic divergence between MLV-like isolates and wild-type strains can be abridged (in terms of minor variants) over a longer time frame or cell passage even though attenuating variants are favoured in cell culture. It would also be interesting to observe MLV quasispecies diversity in the swine model itself as our sample was cell culture propagated. Another obstacle in reversion is that although all differences might be “correctable” via minor SNPs, they would all have to be incorporated into a single haplotype for the wild type strain to re-emerge.

In a majority of the cases, structural ORFs exhibited much higher codon diversity. One-fourth to two-third of all minor variants were capable of causing a change in the respective amino acid. Even after removing the fraction posing deleterious effects to protein functionality, this leaves a significant spectrum for proteomic diversification. Possible reasons for this diversity might have been related to selective pressure imposed by the host immune system (generation of escape variants) or an adaptive response to a new environment (animal to cell culture).

The detection of low frequency deletion mutations in minor variants represented another important finding of the present work since it provided direct insight into one possible mechanism for the emergence and circulation of deletion mutants in the field. Subsequent experiments can now be designed to elucidate the factor(s) that aid(s) the positive selection of such mutants in intra-strain viral populations.

## Supporting Information

Figure S1 **Recombination events detected by the BOOTSCAN method.** Since HK PRRSV strains displayed similar recombination signals, only one from each genotype is shown here while detailed information regarding all signals is given in Table S2 in File S1. Upper panel shows a recombination signal in HK#2 (type 2) with minor parent being PL97-1/LP1. Lower panel shows recombination signal in HK#3 (type 1) with the minor parent being KNU-07. Recombination regions are highlighted in pink.
(DOCX)

File S1 **File containing all supplementary illustrations.**
(DOCX)

Table S1 **Primers used in generating overlapping amplicons spanning PRRSV genomes.**
(DOCX)

Table S2 **Details of recombinational events detected in HK PRRSV strains.**
(DOCX)
