# Supplementary material for: Genomic Evolution of Porcine Reproductive and Respiratory Syndrome Virus (PRRSV) Isolates Revealed by Deep Sequencing
Source: PLoS One. 2014 Apr 3;9(4):e88807. doi: 10.1371/journal.pone.0088807 (PMC3974674; doi:10.1371/journal.pone.0088807)
Supplement: Figure S1 — Recombination events detected by the BOOTSCAN method. Since HK PRRSV strains displayed similar recombination signals, only one from each genotype is shown here while detailed information regarding all signals is given in Table S2 in File S1. Upper panel shows a recombination signal in HK#2 (type 2) with minor parent being PL97-1/LP1. Lower panel shows recombination signal in HK#3 (type 1) with the minor parent being KNU-07. Recombination regions are highlighted in pink. (DOCX) [file pone.0088807.s001.docx]

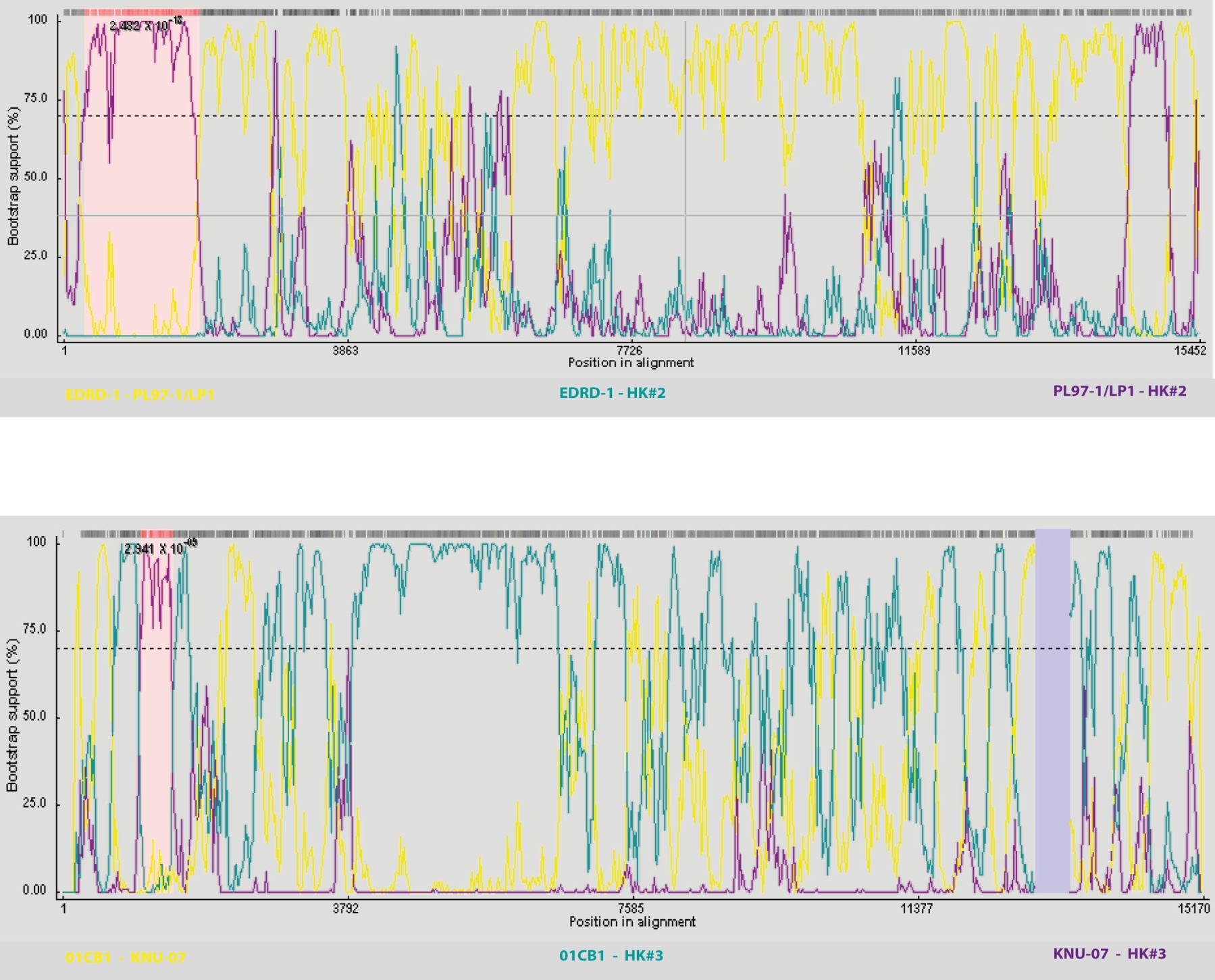


Figure S1 Recombination events detected by the BOOTSCAN method. Since HK PRRSV strains displayed similar recombination signals, only one from each genotype is shown here while detailed information regarding all signals is given in Table S1. Upper panel shows a recombination signal in HK#2 (type 2) with minor parent being PL97-1/LP1. Lower panel shows recombination signal in HK#3 (type 1) with the minor parent being KNU-07. Recombination regions are highlighted in pink.
