# Supplementary material for: Genomic Evolution of Porcine Reproductive and Respiratory Syndrome Virus (PRRSV) Isolates Revealed by Deep Sequencing
Source: PLoS One. 2014 Apr 3;9(4):e88807. doi: 10.1371/journal.pone.0088807 (PMC3974674; doi:10.1371/journal.pone.0088807)
Supplement: File S1 — File containing all supplementary illustrations. (DOCX) [file pone.0088807.s002.docx]

Table S1

| Primer | Genomic position* | Sequence |
| --- | --- | --- |
| Genotype 1 |  |  |
| 1F | 24-40 | 5' CCGCCTGCTGCTCCCTT 3' |
| 1R | 3819-3839 | 5' ACGTGACCCACCGAGTAACTT 3' |
| 2F | 3757-3775 | 5' TTTGGGAACCTGTGCGCGG 3' |
| 2R | 7784-7803 | 5' GCAGACTTGGTGTCTTGAGG 3' |
| 3F | 7682-7701 | 5' AACCTCCAACTCCCTTACAA 3' |
| 3R | 11839-11857 | 5' GTGAATGGCAGAGCGCGAA 3' |
| 4F | 11753-11774 | 5' ATTTTCCTTGCCATACTGTTTG 3' |
| 4R | 14790-14807 | 5' TGTAGAAGTCACGCGAAT 3' |
| Genotype 2 |  |  |
| 1F | 329-345 | 5' AGCCGCTCCGGTGGACG 3' |
| 1R | 4262-4281 | 5' AAGCTCAAAAGAATGAAGGA 3' |
| 2F | 4121-4140 | 5' TGGGTGTATTTTCTGGGTCT 3' |
| 2R | 7681-7700 | 5' TCGCCGTCRACRTTCATCAT 3' |
| 3F | 7447-7466 | 5' GTGTTTTATGAGGAGGTCCA 3' |
| 3R | 11913-11932 | 5' GCRCACGCYAGAATYYTGTA 3' |
| 4F | 11844-11863 | 5' GTCACCCCTTATGAYTACGG 3' |
| 4R | 15278-15298 | 5' TGCGTTGGCAGACTAAACTCC 3' |

*Annealing positions based on GenBank accession entries KF287130 and KF287133 for genotypes 1 and 2 respectively.

Table S2

| Strain | Breakpoint position in alignment | | Major Parent | Minor Parent | P-value from detection program | | | | | | | | |
| --- | --- | --- | --- | --- | --- | --- | --- | --- | --- | --- | --- | --- | --- |
|  | Beginning | Ending |  |  | RDP | GENECONV | Bootscan | Maxchi | Chimaera | SiSscan | PhylPro | LARD | 3Seq |
| #3 | 156 | 466 | EuroPRRSV | 07V063 | 2.419x10^-6^ | 1.59x10^-5^ | 1.025x10^-5^ | NS | NS | 1.833x10^-3^ | NS | NS | NS |
| #3 | 1046 | 1459 | 01CB1 | KNU-07 | 3.193x10^-6^ | NS | 8.324x10^-8^ | 1.754x10^-4^ | 2.521x10^-2^ | NS | NS | NS | 1.585x10^-2^ |
| #3 | 1570 | 2971 | Unknown | HK#5 | 1.806x10^-3^ | NS | 3.192x10^-3^ | 1.072x10^-2^ | 8.7x10^-4^ | 1.803x10^-9^ | NS | NS | NS |
| #3 | 5558 | 6874 | KNU-07 | 07V063 | 2.783x10^-1^ | NS | 5.283x10^-3^ | 8.268x10^-4^ | 1.357x10^-4^ | 1.225x10^-12^ | NS | NS | NS |
| #3 | 11789 | 12263 | LEYPOLYENV | NMEU09-1 | 4.264x10^-7^ | NS | 3.086x10^-6^ | 4.296x10^-3^ | 3.163x10^-3^ | NS | NS | NS | NS |
| #3 | 12817 | 13121 | 01CB1 | NMEU09-1 | 1.506x10^-6^ | NS | 6.667x10^-6^ | 5.331x10^-5^ | 4.04x10^-2^ | NS | NS | NS | NS |
| #5 | 156 | 617 | EuroPRRSV | 07V063 | 2.419x10^-6^ | 1.59x10^-5^ | 1.025x10^-5^ | NS | NS | 1.833x10^-3^ | NS | NS | NS |
| #5 | 928 | 2145 | 01CB1 | KNU-07 | 3.193x10^-6^ | NS | 8.324x10^-8^ | 1.754x10^-4^ | 2.521x10^-2^ | NS | NS | NS | 1.585x10^-2^ |
| #5 | 5552 | 6846 | KNU-07 | 07V063 | 2.783x10^-1^ | NS | 5.283x10^-3^ | 8.268x10^-4^ | 1.357x10^-4^ | 1.225x10^-12^ | NS | NS | NS |
| #5 | 11617 | 12114 | LEYPOLYENV | NMEU09-1 | 4.264x10^-7^ | NS | 3.086x10^-6^ | 4.296x10^-3^ | 3.163x10-3 | NS | NS | NS | NS |
| #5 | 12908 | 13297 | 01CB1 | NMEU09-1 | 1.506x10^-6^ | NS | 6.667x10^-6^ | 5.331x10^-5^ | 4.04x10-2 | NS | NS | NS | NS |
| #8 | 156 | 617 | EuroPRRSV | 07V063 | 2.419x10^-6^ | 1.59x10^-5^ | 1.025x10^-5^ | NS | NS | 1.833x10^-3^ | NS | NS | NS |
| #8 | 984 | 1427 | 01CB1 | KNU-07 | 3.193x10^-6^ | NS | 8.324x10^-8^ | 1.754x10^-4^ | 2.521x10^-2^ | NS | NS | NS | 1.585x10^-2^ |
| #8 | 5913 | 6519 | KNU-07 | 07V063 | 2.783x10^-1^ | NS | 5.283x10^-3^ | 8.268x10^-4^ | 1.357x10^-4^ | 1.225x10^-12^ | NS | NS | NS |
| #10 | 156 | 634 | EuroPRRSV | 07V063 | 2.419x10^-6^ | 1.59x10^-5^ | 1.025x10^-5^ | NS | NS | 1.833x10^-3^ | NS | NS | NS |
| #10 | 1046 | 1427 | 01CB1 | KNU-07 | 3.193x10^-6^ | NS | 8.324x10^-8^ | 1.754x10^-4^ | 2.521x10^-2^ | NS | NS | NS | 1.585x10^-2^ |
| #10 | 5937 | 6663 | HK#5 | HK#3 | 1.195x10^-4^ | 1.121x10^-3^ | 4.738x10^-4^ | 9.06x10^-3^ | NS | 1.088x10^-2^ | NS | NS | NS |
| #10 | 11677 | 13453 | 01CB1 | NMEU09-1 | 1.506x10^-6^ | NS | 6.667x10^-6^ | 5.331x10^-5^ | 4.04x10^-2^ | NS | NS | NS | NS |
| #10 | 13431 | 15128 | Unknown | HKEU16 | 9.063x10^-6^ | 4.762x10^-5^ | 1.161x10^-6^ | NS | NS | 3.117x10^-6^ | NS | NS | NS |
| #2 | 285 | 1836 | EDRD-1 | PL97-1/LP1 | 6.818x10-^14^ | 1.526x10^-2^ | 3.803x10^-15^ | 1.982x10^-5^ | 3.419x10^-4^ | 1.448x10^-7^ | NS | NS | NS |
| #6 | 1 | 361 | HK#16 | HK#12 | 3.946x10^-31^ | 5.338x10^-31^ | 3.507x10^-31^ | NS | NS | 2.305x10^-9^ | NS | NS | NS |
| #6 | 617 | 1470 | EDRD-1 | PL97-1/LP1 | 6.818x10^-14^ | 1.526x10^-2^ | 3.803x10^-15^ | 1.982x10^-5^ | 3.419x10^-4^ | 1.448x10^-7^ | NS | NS | NS |
| #6 | 10877 | 11850 | HK#16 | Unknown | 1.456x10^-17^ | 2.958x10^-8^ | 1.594x10^-10^ | 5.028x10^-4^ | 2.398x10^-5^ | 1.466x10^-3^ | NS | NS | 2.207x10^-11^ |
| #6 | 13590 | 15401 | HK#9 | Unknown | 3.007x10^-25^ | 2.541x10^-21^ | 1.83x10^-24^ | 2.247x10^-16^ | 1.868x10^-17^ | 1.97x10^-18^ | NS | NS | NS |
| #9 | 617 | 1798 | EDRD-1 | PL97-1/LP1 | 6.818x10^-14^ | 1.526x10^-2^ | 3.803x10^-15^ | 1.982x10^-5^ | 3.419x10^-4^ | 1.448x10^-7^ | NS | NS | NS |
| #13 | 988 | 2959 | Unknown | WUH2 | NS | 1.087x10^-2^ | 3.423x10^-4^ | 2.327x10^-2^ | 2.029x10^-2^ | 3.285x10^-3^ | NS | NS | NS |
| #16 | 617 | 1460 | EDRD-1 | PL97-1/LP1 | 6.818x10^-14^ | 1.526x10^-2^ | 3.803x10^-15^ | 1.982x10^-5^ | 3.419x10^-4^ | 1.448x1^0-7^ | NS | NS | NS |

Unknown: recombination event was detected using only the recombinant and a single parent in the alignment.

NS: non-significant


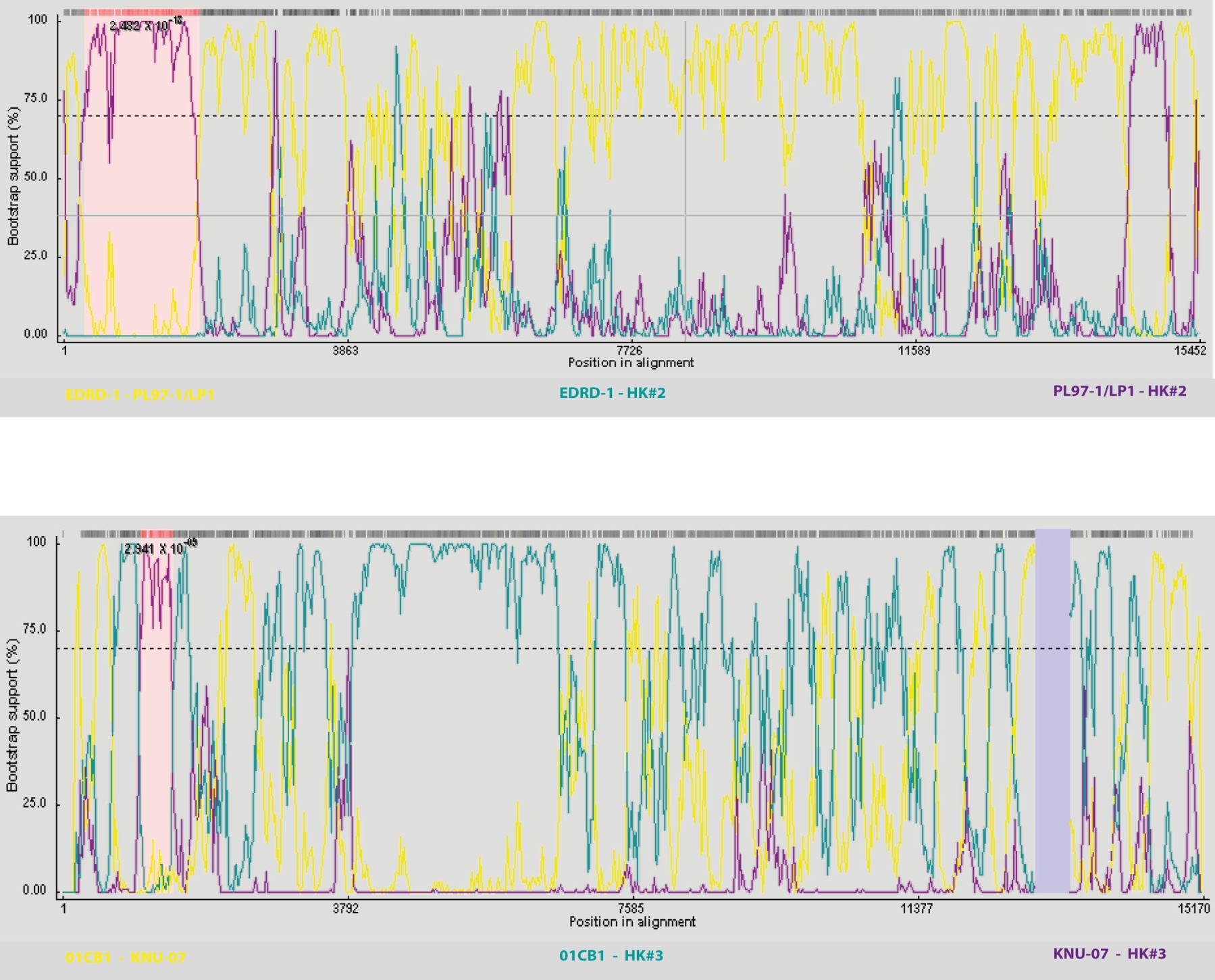


Figure S1
