# Supplementary material for: Genomic Evolution of Porcine Reproductive and Respiratory Syndrome Virus (PRRSV) Isolates Revealed by Deep Sequencing
Source: PLoS One. 2014 Apr 3;9(4):e88807. doi: 10.1371/journal.pone.0088807 (PMC3974674; doi:10.1371/journal.pone.0088807)
Supplement: Table S1 — Primers used in generating overlapping amplicons spanning PRRSV genomes. (DOCX) [file pone.0088807.s003.docx]

Table S1 Primers used in generating overlapping amplicons spanning PRRSV genomes.

| Primer | Genomic position* | Sequence |
| --- | --- | --- |
| Genotype 1 |  |  |
| 1F | 24-40 | 5' CCGCCTGCTGCTCCCTT 3' |
| 1R | 3819-3839 | 5' ACGTGACCCACCGAGTAACTT 3' |
| 2F | 3757-3775 | 5' TTTGGGAACCTGTGCGCGG 3' |
| 2R | 7784-7803 | 5' GCAGACTTGGTGTCTTGAGG 3' |
| 3F | 7682-7701 | 5' AACCTCCAACTCCCTTACAA 3' |
| 3R | 11839-11857 | 5' GTGAATGGCAGAGCGCGAA 3' |
| 4F | 11753-11774 | 5' ATTTTCCTTGCCATACTGTTTG 3' |
| 4R | 14790-14807 | 5' TGTAGAAGTCACGCGAAT 3' |
| Genotype 2 |  |  |
| 1F | 329-345 | 5' AGCCGCTCCGGTGGACG 3' |
| 1R | 4262-4281 | 5' AAGCTCAAAAGAATGAAGGA 3' |
| 2F | 4121-4140 | 5' TGGGTGTATTTTCTGGGTCT 3' |
| 2R | 7681-7700 | 5' TCGCCGTCRACRTTCATCAT 3' |
| 3F | 7447-7466 | 5' GTGTTTTATGAGGAGGTCCA 3' |
| 3R | 11913-11932 | 5' GCRCACGCYAGAATYYTGTA 3' |
| 4F | 11844-11863 | 5' GTCACCCCTTATGAYTACGG 3' |
| 4R | 15278-15298 | 5' TGCGTTGGCAGACTAAACTCC 3' |

*Annealing positions based on GenBank accession entries KF287130 and KF287133 for genotypes 1 and 2 respectively.

Table S2 Details of recombinational events detected in HK PRRSV strains.

| Strain | Breakpoint position in alignment | | Major Parent | Minor Parent | P-value from detection program | | | | | | | | |
| --- | --- | --- | --- | --- | --- | --- | --- | --- | --- | --- | --- | --- | --- |
|  | Beginning | Ending |  |  | RDP | GENECONV | Bootscan | Maxchi | Chimaera | SiSscan | PhylPro | LARD | 3Seq |
| #3 | 156 | 466 | EuroPRRSV | 07V063 | 2.419x10^-6^ | 1.59x10^-5^ | 1.025x10^-5^ | NS | NS | 1.833x10^-3^ | NS | NS | NS |
| #3 | 1046 | 1459 | 01CB1 | KNU-07 | 3.193x10^-6^ | NS | 8.324x10^-8^ | 1.754x10^-4^ | 2.521x10^-2^ | NS | NS | NS | 1.585x10^-2^ |
| #3 | 1570 | 2971 | Unknown | HK#5 | 1.806x10^-3^ | NS | 3.192x10^-3^ | 1.072x10^-2^ | 8.7x10^-4^ | 1.803x10^-9^ | NS | NS | NS |
| #3 | 5558 | 6874 | KNU-07 | 07V063 | 2.783x10^-1^ | NS | 5.283x10^-3^ | 8.268x10^-4^ | 1.357x10^-4^ | 1.225x10^-12^ | NS | NS | NS |
| #3 | 11789 | 12263 | LEYPOLYENV | NMEU09-1 | 4.264x10^-7^ | NS | 3.086x10^-6^ | 4.296x10^-3^ | 3.163x10^-3^ | NS | NS | NS | NS |
| #3 | 12817 | 13121 | 01CB1 | NMEU09-1 | 1.506x10^-6^ | NS | 6.667x10^-6^ | 5.331x10^-5^ | 4.04x10^-2^ | NS | NS | NS | NS |
| #5 | 156 | 617 | EuroPRRSV | 07V063 | 2.419x10^-6^ | 1.59x10^-5^ | 1.025x10^-5^ | NS | NS | 1.833x10^-3^ | NS | NS | NS |
| #5 | 928 | 2145 | 01CB1 | KNU-07 | 3.193x10^-6^ | NS | 8.324x10^-8^ | 1.754x10^-4^ | 2.521x10^-2^ | NS | NS | NS | 1.585x10^-2^ |
| #5 | 5552 | 6846 | KNU-07 | 07V063 | 2.783x10^-1^ | NS | 5.283x10^-3^ | 8.268x10^-4^ | 1.357x10^-4^ | 1.225x10^-12^ | NS | NS | NS |
| #5 | 11617 | 12114 | LEYPOLYENV | NMEU09-1 | 4.264x10^-7^ | NS | 3.086x10^-6^ | 4.296x10^-3^ | 3.163x10-3 | NS | NS | NS | NS |
| #5 | 12908 | 13297 | 01CB1 | NMEU09-1 | 1.506x10^-6^ | NS | 6.667x10^-6^ | 5.331x10^-5^ | 4.04x10-2 | NS | NS | NS | NS |
| #8 | 156 | 617 | EuroPRRSV | 07V063 | 2.419x10^-6^ | 1.59x10^-5^ | 1.025x10^-5^ | NS | NS | 1.833x10^-3^ | NS | NS | NS |
| #8 | 984 | 1427 | 01CB1 | KNU-07 | 3.193x10^-6^ | NS | 8.324x10^-8^ | 1.754x10^-4^ | 2.521x10^-2^ | NS | NS | NS | 1.585x10^-2^ |
| #8 | 5913 | 6519 | KNU-07 | 07V063 | 2.783x10^-1^ | NS | 5.283x10^-3^ | 8.268x10^-4^ | 1.357x10^-4^ | 1.225x10^-12^ | NS | NS | NS |
| #10 | 156 | 634 | EuroPRRSV | 07V063 | 2.419x10^-6^ | 1.59x10^-5^ | 1.025x10^-5^ | NS | NS | 1.833x10^-3^ | NS | NS | NS |
| #10 | 1046 | 1427 | 01CB1 | KNU-07 | 3.193x10^-6^ | NS | 8.324x10^-8^ | 1.754x10^-4^ | 2.521x10^-2^ | NS | NS | NS | 1.585x10^-2^ |
| #10 | 5937 | 6663 | HK#5 | HK#3 | 1.195x10^-4^ | 1.121x10^-3^ | 4.738x10^-4^ | 9.06x10^-3^ | NS | 1.088x10^-2^ | NS | NS | NS |
| #10 | 11677 | 13453 | 01CB1 | NMEU09-1 | 1.506x10^-6^ | NS | 6.667x10^-6^ | 5.331x10^-5^ | 4.04x10^-2^ | NS | NS | NS | NS |
| #10 | 13431 | 15128 | Unknown | HKEU16 | 9.063x10^-6^ | 4.762x10^-5^ | 1.161x10^-6^ | NS | NS | 3.117x10^-6^ | NS | NS | NS |
| #2 | 285 | 1836 | EDRD-1 | PL97-1/LP1 | 6.818x10-^14^ | 1.526x10^-2^ | 3.803x10^-15^ | 1.982x10^-5^ | 3.419x10^-4^ | 1.448x10^-7^ | NS | NS | NS |
| #6 | 1 | 361 | HK#16 | HK#12 | 3.946x10^-31^ | 5.338x10^-31^ | 3.507x10^-31^ | NS | NS | 2.305x10^-9^ | NS | NS | NS |
| #6 | 617 | 1470 | EDRD-1 | PL97-1/LP1 | 6.818x10^-14^ | 1.526x10^-2^ | 3.803x10^-15^ | 1.982x10^-5^ | 3.419x10^-4^ | 1.448x10^-7^ | NS | NS | NS |
| #6 | 10877 | 11850 | HK#16 | Unknown | 1.456x10^-17^ | 2.958x10^-8^ | 1.594x10^-10^ | 5.028x10^-4^ | 2.398x10^-5^ | 1.466x10^-3^ | NS | NS | 2.207x10^-11^ |
| #6 | 13590 | 15401 | HK#9 | Unknown | 3.007x10^-25^ | 2.541x10^-21^ | 1.83x10^-24^ | 2.247x10^-16^ | 1.868x10^-17^ | 1.97x10^-18^ | NS | NS | NS |
| #9 | 617 | 1798 | EDRD-1 | PL97-1/LP1 | 6.818x10^-14^ | 1.526x10^-2^ | 3.803x10^-15^ | 1.982x10^-5^ | 3.419x10^-4^ | 1.448x10^-7^ | NS | NS | NS |
| #13 | 988 | 2959 | Unknown | WUH2 | NS | 1.087x10^-2^ | 3.423x10^-4^ | 2.327x10^-2^ | 2.029x10^-2^ | 3.285x10^-3^ | NS | NS | NS |
| #16 | 617 | 1460 | EDRD-1 | PL97-1/LP1 | 6.818x10^-14^ | 1.526x10^-2^ | 3.803x10^-15^ | 1.982x10^-5^ | 3.419x10^-4^ | 1.448x1^0-7^ | NS | NS | NS |

Unknown: recombination event was detected using only the recombinant and a single parent in the alignment.

NS: non-significant


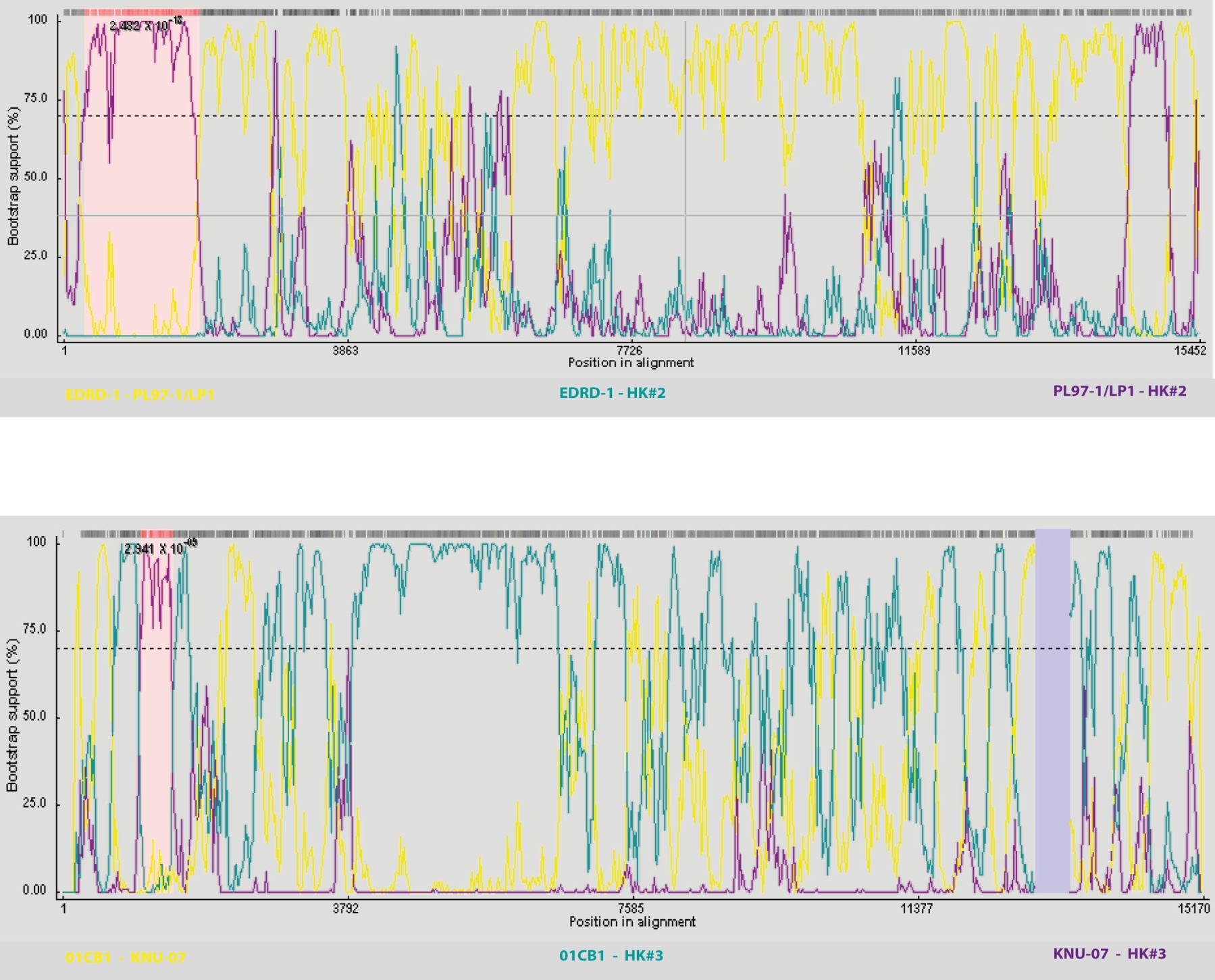


Figure S1 Recombination events detected by the BOOTSCAN method. Since HK PRRSV strains displayed similar recombination signals, only one from each genotype is shown here while detailed information regarding all signals is given in Table S1. Upper panel shows a recombination signal in HK#2 (type 2) with minor parent being PL97-1/LP1. Lower panel shows recombination signal in HK#3 (type 1) with the minor parent being KNU-07. Recombination regions are highlighted in pink.
